# Awareness, Self-Use, Perceptions, Beliefs, and Attitudes toward Complementary and Alternative Medicines (CAM) among Health Professional Students in King Saud bin Abdulaziz University for Health Sciences Jeddah, Saudi Arabia

**DOI:** 10.1155/2020/7872819

**Published:** 2020-04-21

**Authors:** Aslam Khan, Mohamed Eldigre Ahmed, Ahmed Aldarmahi, Syed Faisal Zaidi, Ahmad M. Subahi, Adnan Al Shaikh, Zackary Alghamdy, Lujain Ali Alhakami

**Affiliations:** ^1^College of Science and Health Professions, King Saud Bin Abdulaziz University for Health Sciences (KSAU-HS), Ministry of National Guard-Health Affairs (MNG-HA), Jeddah, Saudi Arabia; ^2^King Abdullah International Medical Research Center, Jeddah, Saudi Arabia; ^3^College of Medicine, King Saud Bin Abdulaziz University for Health Sciences (KSAU-HS), Ministry of National Guard-Health Affairs (MNG-HA), Jeddah, Saudi Arabia

## Abstract

**Background:**

Around 75% of the world's population relies on the use of complementary and alternative medicines (CAM) for their healthcare. Thus, we aimed at assessing the attitude and perception of health professional students about CAM and correlate the results with their demographical data.

**Methods:**

An observational cross-sectional study was conducted at King Saud bin Abdulaziz University for Health Sciences (KSAU-HS), Jeddah, Saudi Arabia, by enrolling 350 students. A self-administered questionnaire was used for data collection. Descriptive and inferential statistical analysis was performed using SPSS.

**Results:**

Results of our data suggested that majority of students were aware of the CAM modalities and considered prayers/spirituality (83.6%), massage (72.5%), nutritional supplements (74.1%), cupping (68.5%), herbal medicine (66.2), and yoga (61.6%) as most effective and least harmful CAM modalities. The students acquired more information about CAM from media (55%), books (56%), friends/relatives (59.7%), and other health professionals (58.4%), however, very little information from formal CAM courses or training (36.7%), which shows the lack of courses and trained health professional in the field of CAM. Despite utilizing nonreliable sources of CAM information, the majority of students had positive attitudes and perceptions about CAM usage. Majority of the students (75.8%) believe in the use of CAM for the mental and spiritual aspect of health and think that CAM providers give good information on maintaining a healthy lifestyle. The data also showed a significant (*P* < 0.05) association between awareness about CAM and gender. A significantly higher percentage of female students were using yoga and aromatherapy, while cupping was mostly used by male students. Similarly, a significant association was found among the different colleges and level of students in most of the CAM modalities. However, no association was found between the awareness and use of CAM with the educational level of parents or relatives in health sector except for acupuncture and cupping, where student's awareness about acupuncture and cupping was found to have a significant positive association with mother educational level. On the other hand, a significant inverse association was found between acupuncture, yoga, cupping, and aromatherapy with family income.

**Conclusion:**

In conclusion, despite the lack of proper courses and professionally trained healthcare providers in the field of CAM, students demonstrated positive attitudes and beliefs toward the safety and effectiveness of CAM.

## 1. Introduction

Traditional medicine (TM) has a long history, and World Health Organization (WHO) defines it as “the sum total of the knowledge, skills, and practices based on the theories, beliefs, and experiences indigenous to different cultures, whether explicable or not, used in the maintenance of health as well as in the prevention, diagnosis, improvement, or treatment of physical and mental illness.” The term “complementary medicine” or “alternative medicine” refers to a broad set of healthcare practices that are not part of that country's own tradition or conventional medicine and are not fully integrated into the dominant healthcare system. They are used interchangeably with traditional medicine in some countries. However, complementary and alternative medicine (CAM) is also often termed as “traditional medicine (TM)” or “nonconventional medicine.” CAM refers to local knowledge, belief systems, and therapeutic practices that are used in different countries for the treatment or prevention of different diseases, especially chronic diseases and products that are not generally considered part of conventional (Western or allopathic) medicine [1]. The evolution of different systems of traditional medicine emerged as a result of the traditional knowledge of using herbs for various disorders. According to the World Health Organization (WHO), “health is not merely the absence of disease or infirmity but a state of complete physical, mental, and social well-being” [2]. Conventional (Western) medicine focuses mainly on the physical health and/or prevention/treatment of diseases but focuses little on the mental, social, and spiritual aspects of health. Therefore, National Institute of Health (NIH) has established National Center for the Complementary and Integrative Health (NCCIH) in order to provide funds and conduct research to fill the gap between research and health information regarding CAM therapies, especially for Integrative Health Care which combines both conventional and complementary approaches, for which there is evidence of effectiveness, in a coordinated way. This approach to health and wellness is growing across the US. Researchers are currently exploring the potential benefits of integrative health in a variety of situations, such as pain management in military personnel and veterans, relief of symptoms in cancer patients and survivors, and programs to promote healthy behaviours [3].

Globally, CAM is a multibillion-dollar industry and has achieved exponential growth in the last two decades in industrialized countries [4]. Recent research is now showing a remarkable increase in support for, and usage of, therapeutic practices outside mainstream medicine [5]. According to the WHO, plants provide an economical and affordable source of drugs for three-quarters of the world population and its therapeutic use is increasing day by day [6]. Herbal medicines are considered to be safer and economical sources of drugs and also contain synergistic and/or adverse effects neutralizing potential and they act through multiple pathways [4].

The use of CAM has persisted for centuries worldwide. According to the WHO survey, traditional healers treat 90% in Bangladesh, 85% in Burma, 80% in India, 75% in Nepal, 65% patients in Sri Lanka, and 60% in Indonesia [7]. Pakistan has a long history of traditional medicine where there are 39,584 Hakims/Tabibs (traditional medicine healers) and 90,000 homoeopathic doctors [8], while in Saudi Arabia, there is hardly a city or village in the country where traditional medicines are not used or sold. They are also commonly used in home remedies for certain ailments [9]. Also, in Saudi Arabia, traditional medicines, most commonly spiritual healing and herbal remedies, are widely used. However, there was official resistance to complementary/alternative medicine until the 1990s when more Saudi Arabians demanded access to complementary/alternative medicine, and some professionals who had been trained abroad began to practice [9]. The current, detailed, and comprehensive picture of CAM usage by Saudis is not yet clear; however, studies from different regions of Saudi Arabia also indicate the wide use of CAM in Saudi Arabia. In 2000, a study in the capital city Riyadh showed that 46% of the population used CAM, and 19% of them used CAM in the previous 12 months [10]. Another household survey conducted in 2008 in Riyadh region found that 73% of the population had previously used CAM, and 67% used it in the last year [11]. A study conducted in Qassim Province in 2011 showed a total of 74% of subjects had visited CAM providers in 12 months before the survey. This percentage decreased to 47.6% when spiritual healers were excluded [12].

There is a clear evidence of the revival of interest and use in CAM practices, especially herbal medicine. The sales of herbal products in the world are worth staggering over 100 billion dollars a year [4]. In the European community, the annual sales of the herbal medicine are around $7 billion, while in the USA, the sale of herbal products has tremendously increased from $200 million in 1988 to >$3.3 billion in 1997 [13]. In spite of such a high dependency, little scientific research has been carried out on traditional medicines. “This gap between the use of traditional remedies and their scientific basis exists mainly due to the lack of active interactions between the modern health professionals and traditional healers,” lack of modern testing technology, and a shortage of qualified scientists in the field of natural products pharmacology. However, pharmaceutical research centers, universities, and even pharmaceutical companies are filling this gap and bringing together such teams and focus research on phytomedicine [14, 15].

Pharmacopoeias of several countries are full of a huge amount of medicinal plants being used for the treatment of various health diseases, especially chronic ailments. In recent years, there has been a revival of interest in the natural product for different illnesses and are considered being safe, effective, and culturally acceptable. Therefore, the aim of this study is to know and measure the health professional student's perception, knowledge, understanding, and use of CAM and also to describe the factors that are associated with their attitude and possible reasons to use CAM.

## 2. Methodology

### 2.1. Study Design and Sampling

This is a descriptive observational cross-sectional survey, where multiple-choice questionnaires were used to target health science students at King Saud bin Abdulaziz University for Health Sciences (KSAU-HS), Jeddah, Saudi Arabia, from December 2018 to July 2019. The university branch in Jeddah was established in 2012 and consists of four colleges, including the College of Science and Health Professions (COSHP), College of Medicine (COM), College of Applied Medical Sciences (CAMS), and College of Nursing (CON).

The questionnaires were distributed among the selected students together with a written consent form that explained the purpose of the research and assured them of confidentiality. After that, the students were allowed to fill the questionnaire and one of the investigators was there to answer any query.

### 2.2. Development of the Questionnaire

A self-administered questionnaire was used in this survey. The questionnaire was designed after extensive literature review [16–28]. The questionnaire was validated by performing face validity from expert biostatisticians and health professionals. The content validity was done by a subject expert. Eventually, a pilot study was carried out to check for the clarity of the question and calculating Cronbach's alpha for reliability. The value for Cronbach's alpha for reliability coefficient was computed at 0.84, indicating a reasonable level of reliability of responses. Modifications were made in the questionnaire according to the feedback from the pilot testing. Arabic translation was checked by an Arabic language expert.

The questionnaire contained six parts, the first part is demographic characteristics, the second part is awareness and use of CAM, the third part is the perception of effectiveness and safety of CAM, the fourth part is sources of information, the fifth part is perceived barriers to CAM, and the sixth part is beliefs and attitudes toward CAM. The questionnaire was bilingual and written in English and then translated to Arabic. This is due to the fact that Arabic is the national language of Saudi Arabia and some of the English terms for CAM might be unfamiliar to the student. After the assortment of the data collection form, the data were saved in a personal computer with a password, so no one can access it. Privacy and confidentiality of the participants were completely protected, and no direct identification was used. Research data, both soft and hard copies, were maintained in a secure location and/or unit within MNG-HA premises and were only be accessible by the research team.

### 2.3. Estimated Sample Size

The total number of students in COSHP, COM, and COAMS is about 2243, where 1154 (51%) are male students and 1089 (49%) are female students. All students in the university were eligible to be included in the study except for the CON, where all students are females. The sample size (*n* = 385) was determined by using the Cochran equation (1977) [29] of simple random sampling which depends on the population proportion, as given below. Random stratified sampling was assigned where colleges were considered to be the strata and samples were collected based on the population proportion of each college. 385 questionnaires were distributed; however, 350 students have successfully completed the study.

(1) $$\begin{array}{c} n=\frac{z^{2}*P*\left(1-P\right)}{d^{2}}, \end{array}$$

where *n* is the sample size, *z* the standard variable of the normal distribution corresponding to 95% confidence level, *P* the anticipated population proportion of knowledge about CAM, *d* the absolute statistical precision on either side of the anticipated population proportion by taking *P*=50% and *d* = 0.05; then, the initial sample size will be *n* = 1.96^2^ ∗ 0.5 ∗ 0.5/0.0025 = 385.

### 2.4. Statistical Analysis

Descriptive and inferential statistical analysis was performed using SPSS version 25. Descriptive statistics including frequency tables, graphs, and means were conducted for data presentation. Inferential methods included chi-square test for independence and were conducted to test the association between the demographic and basic characteristics of students and the awareness and use of CAM. All hypotheses were tested at the 5% level of significance.

## 3. Results

A total of 385 questionnaires were distributed to the students, out of which 350 students responded with written consent to fill questionnaires while 35 did not consent to fill the questionnaire, making an overall response rate of 90.9%.

### 3.1. Basic Information

The basic information of students in the sample is summarized in Table 1. Male students constitute 51.4% of the total sample, while female students constitute 48.6% of the sample size. The samples were collected based on the population size of the respective colleges; 50% were from COSHP, 36.3% were from COM while 13.7% were from the CAMS. As a whole, about 38.6% of the students were in their fourth or above educational level. The results on the level of parents' education revealed that 73.4% of the student's fathers had a university or above educational level, and about 66.6% of student's mothers had a university or above educational level. Regarding the employment status of parents, 3.4% of the student's fathers were unemployed, while the percentage of unemployment among student's mothers is about 44.9%. Around 60% of the student's parents have a monthly income of more than SR 20,000. The percentage of students who had a family member related to the healthcare sector is 46.3%.

### 3.2. Awareness, Self-Use, Effectiveness, and Harmfulness of CAM Modalities

Results of our study show that prayers and spirituality had the highest percentage (83%) of awareness among students followed by massage and nutritional supplements. On the other hand, bloodletting followed by aromatherapy had the lowest level of awareness. Regarding the use of CAM modalities, about 70.0% of students have used prayers and spirituality in their lives, and the use of bloodletting and aromatherapy was lowest (Table 2).

Effectiveness and harmfulness of CAM modalities are given as percentages of Likert scale mean according to the student's perception. The results showed that the highest percentage of effectiveness of CAM modalities was associated with prayers and spirituality followed by nutritional supplements and massage. Bloodletting followed by aromatherapy was found to have the lowest percentages regarding the effectiveness of CAM modalities (Table 2; column 3).

Student's opinions on the harmfulness of CAM modalities show that bloodletting followed by acupuncture has the highest of harmfulness, while prayers/spirituality and massage were considered as the lowest harmful CAM modality (Table 2; column 4).

### 3.3. Sources of CAM Information

Table 3 illustrates the sources of information about CAM acquired by the same sample of university students. A higher percentage of students considered friends and relatives as the primary source of information about CAM. The second source of information about CAM was health professionals. Students obtained the least information about CAM from any formal CAM course or training.

### 3.4. Perceived Barriers to CAM Implementation

Table 4 represents the students' opinions and perception about the barriers and restrictions of CAM use and implementation. The majority of the students considered the lack of knowledge about CAM as the primary obstacle to CAM use and implementation. The second and third barriers of CAM implementation according to students in the sample were lack of scientific evidence and lack of trained professionals, respectively.

### 3.5. Beliefs and Attitudes toward CAM

Table 5 represents the Likert scale mean values of the response of student's beliefs and attitudes toward CAM. Exceeding the midpoint (2.5) represents a positive belief. Data demonstrated most of the students have a positive score toward all the six questions reflecting generally good beliefs and attitudes toward CAM among the participating students irrespective of the gender and colleges. However, a significantly higher number of female students respond positivity to the statement “People are more likely to use CAM if his/her teachers discuss it with them.”

### 3.6. The Association between Gender and Awareness and Use of CAM Modalities

Table 6 summarizes the results of the chi-square test for independence between students' gender and awareness and use of CAM modalities. A significant association was found between gender of students and awareness about CAM modalities except for cupping practice, and female students were noticed to have significantly more awareness compared to males. For the association between gender and use of CAM modalities, a significant relationship was found between gender and use of yoga, cupping, and aromatherapy. The percentage of using modalities showed that 21.2% of female students used yoga, compared to 9.5% of male students. Cupping practice was more spread among male students, while female students are using yoga and aromatherapy more than male students.

### 3.7. The Association between College and Awareness and Use of CAM Modalities

Results of the chi-square test for association between student's college and awareness and use of CAM modalities are summarized in Table 7. A strong association was found between the type of college and CAM modalities awareness, except for cupping, bloodletting, and prayers. Percentages of awareness among COM students were higher followed by COSHP students. CAMS students were found to have the lowest percentage of awareness. The use of acupuncture, cupping, herbal medicine, and nutritional supplements was significantly associated with the type of student's college. The results showed that the awareness and use of prayer/spirituality were much higher than other CAM modalities in all the students across all the colleges.

### 3.8. Association between the Level of College and Awareness and Use of CAM Modalities

The association between the level of students and awareness and use of CAM modalities is shown in Table 8. Except for cupping, bloodletting, and prayers, a strong association was found between student's level and awareness; third level students were found to have the highest percentages of awareness.

### 3.9. Association between Father Education and Awareness and Use of CAM Modalities

Table 9 summarizes the results of association test between father educational level and student's awareness and use of CAM modalities. It can be seen from the table that no significant association was found between father educational level and students' awareness about all the mentioned CAM modalities as indicated by the test *P* values. Regarding the use of CAM modalities, the only significant association was found between father education and student's use of prayers and spirituality as a CAM modality.

### 3.10. Association between Mother Education and Awareness and Use of CAM Modalities

The association between mother education and student's awareness and use of CAM modalities depicted in Table 10. Student's awareness about acupuncture and cupping was found to have a significant association with mother educational level; the higher the educational level of mothers, the higher the percentage of awareness. The use of bloodletting was considered as the only modality associated with mother education.

### 3.11. Association between Having Relatives in the Health Sector and Awareness and Use of CAM Modalities

Table 11 summarizes the results of the test of association between having relatives in the health sector and student's awareness and use of CAM modalities. It is clear from the table that having a relative in the health sector was only associated with student's awareness about prayers and spirituality as CAM modality. No significant relationship was revealed between having a relative in the health sector and student's use of CAM modalities.

### 3.12. Association between Income and Awareness and Use of CAM Modalities

Table 12 represents the association between family income and awareness and use of CAM. The results show no significant difference between family income and awareness of student except nutritional supplements, which was significantly associated with family income. Students with high-income families were found to be more aware about the nutritional supplements, whereas students with low-income families were higher in using acupuncture, yoga, and cupping. However, aromatherapy has been used significantly by students belong to higher-income families.

## 4. Discussion

The data and results of our study revealed that prayer/spirituality is the most widely recognized and effective modality with minimum harm among all the students, followed by massage, nutritional supplements, cupping, yoga, herbal medicine, acupuncture, and aromatherapy, whereas bloodletting was the least commonly recognized and effective while considered as the most harmful modality. Regarding the use and effectiveness of prayers and massage, the same pattern is seen in the previous studies, done throughout the world, which demonstrates that prayers/spiritualty and massage are the most commonly known CAM practices among students in Pakistan [30], Ethiopia [31], Kuwait [32], Syria [24], Ghana [33], and Czech Republic [34]. Our data also suggest that only a few (less than 35%) percent of the students have used massage, nutritional supplements, cupping, and yoga; however, still most of the students (>60%) think that these modalities are effective, but may cause harm upto some extent. Similarly, less than 5% of the students have never used acupuncture, aromatherapy, or bloodletting; however, more than 38% and 40% of the students were aware of their effectiveness and harmfulness, respectively. The high percentage of awareness, self-use, and effectiveness of prayers/spirituality, massage, and cupping may be associated with religious and cultural norms being practiced by the elders who inculcated the beliefs of safety and efficacy of these modalities in their decedents by communicating personal experiences and exposing their children to practicing professionals [30]. Therefore, we investigated the sources of their information and found that most of the students received information about the CAM from their relatives/friends, other health professionals, books and media, and CAM practitioners and least information from CAM courses or training, which shows the lack of formal courses and training in the colleges/universities. However, most of the students think that lack of trained professionals, lack of scientific evidence, long time for treatment, and lack of knowledge about CAM could be the barriers in the implementation and use of CAM. Therefore, looking into widespread use and practice of CAM in Saudi Arabia [35], modern CAM practices should also be introduced in the Saudi community through well-established clinics employing acupuncture which is mostly practiced in the private sector. The Saudi Ministry of Health has established a center for CAM by ministerial decree (no. 236) date 10/8/1429 H (12/8/2008 G) to regulate CAM practices within the healthcare services and to use evidence-based CAM as complementary to conventional medicine [35].

In addition, we also look into the association among the awareness and use of CAM with the gender, colleges, level of study, education of father, education of mother, the income of parents, and relatives/friends in the health sector of the students and found some interesting results. Up to the best of our knowledge, this is the first study among health professional students, finding the correlation among awareness and use of CAM with all these mentioned parameters.

Our study shows that the overall awareness of female students is significantly higher than that of male students, except for cupping where the awareness of the male and female students is comparable. There is no significant difference between the male and female students in terms of “self-use” of most of the CAM modalities except cupping, which is used significantly higher by males than females while yoga and aromatherapy are used significantly higher by female students than male students. The Arabic name for cupping is “Hijama” and one of the popular and mostly used CAM modalities in Saudi Arabia [36]. It is an ancient holistic method used for the treatment of various diseases. The reason for significantly lesser use by the females as compared to males may be due to its effect on the skin, such as skin pinching, and/or its contraindication in pregnant and menstruating woman [37]. Our study also shows that a significantly higher percentage of female students are more aware and use yoga and aromatherapy than male students, This is in line with the previous study in UAE which shows that males prefer competitive activities such as football, fitness/weights, and jogging while females prefer activities with people of the same gender and activity types such as aerobics, cycling, walking, and yoga [38].

In order to know about the sources of information about the CAM, our data show that students get most of the information from their relatives or friends, books, and media. This shows the influence of family and culture [39] and mass media as can be seen in previous studies from Pakistan [40] and Majmaah Province of Saudi Arabia [41]. However, this information can be misleading and our data show that most of the students consider lack of trained professionals, lack of scientific evidence, and lack of knowledge about CAM, as indicated in previous studies [32, 42, 43].

A large number of evidence-based studies suggested that students from Pakistan, United States, Saudi Arabia, and Malaysia exhibited positive attitudes and beliefs toward CAM usage [30, 41, 44, 45]. This is in line with our study results which indicate that more than 60% of the students have shown that overall CAM providers give good information on maintaining a healthy lifestyle and encourage the use of CAM for the mental and spiritual aspect of health. However, as indicated in our study results, students believe that they will use CAM more likely if they have been given more knowledge by health professionals and teachers about CAM. This suggests the importance and inclusion of formal CAM courses in the curriculum, which will benefit the community in both the use and misuse of CAM and will highlight the need to revise the academic curricula and health policies to regulate and standardize healthcare practices of CAM to ensure public protection [30].

A significant association was found between the type of college/level of education and awareness/self-use of CAM modalities. Medical college students had a higher level of awareness than other college students; however, the use of CAM modalities was lower in medical college students than students of other colleges, except for prayer/spirituality and nutritional supplements. The reason could be the lack of trust in CAM modalities of medical students due to insufficient information about the risks and benefits of CAM [46].

In our study, we could not find any significant association between father/mother education or relatives in the health sector and awareness/self-use of most of the CAM modalities; however, a significant association was found between the families' monthly income and self-use of acupuncture, yoga, cupping, and aromatherapy. This could be due to the different socioeconomic backgrounds of the students where they come from and the sources of knowledge available in their respective areas. Also, this could be especially true, when we consider the high percentage of students who acquire knowledge on CAM mainly through their relatives and friends [47].

### 4.1. Strength

This is the first study accessing the awareness, self-use, perceptions, beliefs, and attitude toward CAM among health professional students in Jeddah, Saudi Arabia, using a validated questionnaire and looking into the association of student's demographic characteristics with their awareness self-use of different CAM modalities.

### 4.2. Limitations and Recommendations

Our study has certain limitations as follows. Despite the good response rate that we received, our sample size was relatively smaller for cross-sectional studies on awareness, attitudes, and use. As this is a cross-sectional study design, thus factors affecting student's responses cannot be studied over time. Additionally, the knowledge, as well as attitudes of the students regarding CAM, may change over time, as also indicated in the previous studies [30]; therefore, a longitudinal study should be performed to better explain these behaviours. Another limitation was the single center, i.e., King Saud bin Abdulaziz University for Health Sciences, Jeddah, Saudi Arabia, which cannot be generalized to other universities of Jeddah or Saudi Arabia, since the university is specialized in health sciences. Therefore, more studies are required from different universities and also in the general population and healthcare professionals which might have different opinions.

## 5. Conclusion

In conclusion, a high percentage of students demonstrated awareness, effectiveness, and positive attitudes toward most of the CAM modalities. They exhibited better awareness of CAM and consider spiritual healing/prayer, massage, yoga, nutritional supplements, and cupping as the most effective and least harmful CAM modalities. Therefore, we suggest that formal courses and training should be included in the curriculum of health professional students to bridge their knowledge gap and acquire the necessary tools to meet the patients' expectations and needs in relation to CAM use.

## Figures and Tables

**Table 1:** Basic demographic information of the population.

Variable	*N* = 350	%
Gender		
Male	180	51.4
Female	170	48.6

College		
COSHP	175	50.0
COM	127	36.3
CAMS	48	13.7

Level/grade		
First	58	16.6
Second	51	14.6
Third	106	30.3
Fourth and above	135	38.6

Father education		
None	4	1.1
Primary	14	4.0
Secondary	75	21.4
University	257	73.4

Mother education		
None	11	3.1
Primary	24	6.9
Secondary	82	23.4
University	233	66.6

Father employment		
Employed	168	48.0
Self-employed	57	16.3
Retired	113	32.3
Unemployed	12	3.4

Mother employment		
Employed	109	31.1
Self-employed	20	5.7
Retired	64	18.3
Unemployed	157	44.9

Monthly income (SR)		
<5000	15	4.3
5000–15000	128	36.6
>20000	207	59.1

Family-related to healthcare		
Yes	162	46.3
No	188	53.7

CAMS: College of Applied Medical Sciences; COM: College of Medicine; COSHP: College of Science and Health Professions.

**Table 2:** Percentages of awareness, self-use, effectiveness, and harmfulness of CAM modalities.

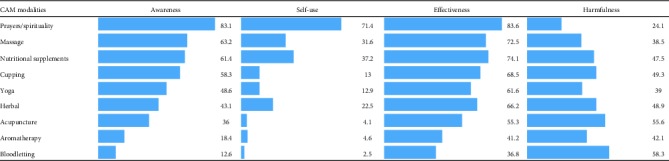

**Table 3:** Sources of information about CAM.

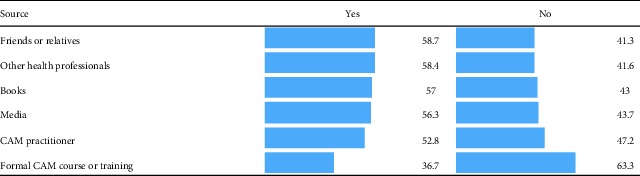

**Table 4:** Means and agreement percentage of perceived barriers to CAM implementation.

Statement	Scale mean	% agreement
Lack of trained professionals	3.91	78.2
Lack of scientific evidence	3.92	78.4
Long time for treatment	3.55	71.0
Lack of knowledge about CAM	4.12	82.4

**Table 5:** Means of values (out of 5) on beliefs and attitudes toward CAM.

Statement	Overall		Gender			Colleges			
	Out of 5	%	Male	Female	*P* value	COSHP	COM	CAMS	*P* value
(1) CAM providers give good information on maintaining a healthy lifestyle	3.31	66.2	3.27	3.38	0.399	3.34	3.26	3.52	0.467
(2) CAM involves natural plant formulas which are more healthy than taking drugs given by the medical doctor	2.91	58.2	2.94	3.32	0.045	3.1	2.73	**3.52**	**0.001**
(3). The more the knowledge a person has about CAM, the more likely he/she is to use it	3.62	72.4	3.66	3.51	0.197	**3.71**	3.59	3.56	0.603
(4) People are more likely to use CAM if his/her friends or relatives discuss it with them	3.8	76	3.77	3.88	0.368	3.83	**3.87**	3.29	**0.014**
(5) People are more likely to use CAM if his/her teachers discuss it with them	3.86	77.2	3.77	**4.07**	**0.025**	3.81	**3.94**	3.41	**0.04**
(6) Believing on mental and spiritual aspect of health encourages the use of CAM	3.79	75.8	3.75	3.87	0.411	3.64	3.88	3.56	0.147

Number represents the mean scale out of 5.*P* < 0.05 considered to be significant.

**Table 6:** The association of gender with awareness and use of CAM modalities.

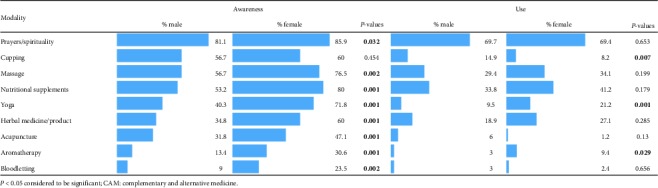

**Table 7:** The association of college with awareness and use of CAM modalities.

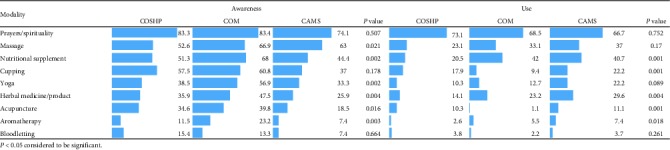

**Table 8:** The association of the level of college with awareness and use of CAM modalities.

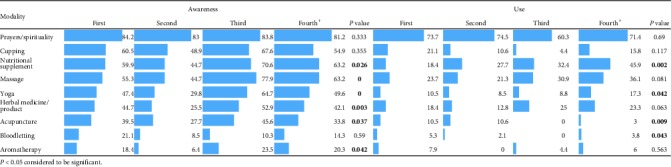

**Table 9:** Association between father education and awareness and use of CAM modalities.

Modality	Awareness					Use				
	None	Primary	Secondary	University	*P* value	None	Primary	Secondary	University	*P* value
Acupuncture	25.0	9.1	30.8	39.8	0.191	25.0	0.0	3.1	4.9	0.213
Yoga	100.0	36.4	43.3	51.5	0.297	50.0	0.0	12.3	13.1	0.248
Cupping	75.0	45.5	56.9	58.3	0.643	25.0	27.3	13.8	11.7	0.509
Aromatherapy	25.0	18.2	16.9	18.9	0.948	0.0	9.1	3.1	5.3	0.514
Bloodletting	0.0	9.1	13.8	13.6	0.892	0.0	0.0	3.1	2.9	0.886
Herbal medicine/product	50.0	36.4	53.8	38.8	0.243	25.0	45.5	30.8	17.0	0.085
Massage	75.0	63.6	63.1	62.1	0.997	0.0	9.1	27.7	33.5	0.240
Nutritional supplements	75.0	45.5	60.0	62.1	0.757	25.0	18.2	35.4	37.4	0.782
Prayers/spirituality	100.0	100.0	80.0	82.0	0.483	50.0	100.0	63.1	70.4	0.047

*P* < 0.05 considered to be significant.

**Table 10:** Association between mother education and awareness and use of CAM modalities.

Modality	Awareness					Use				
	None	Primary	Secondary	University	*P* value	None	Primary	Secondary	University	*P* value
Acupuncture	12.5	13.0	38.7	**39.4**	**0.035**	12.5	8.7	3.2	4.1	0.154
Yoga	62.5	26.1	50.0	51.8	0.360	25.0	17.4	9.7	13.0	0.276
Cupping	37.5	52.2	**72.6**	54.4	**0.005**	12.5	21.7	19.4	9.8	0.218
Aromatherapy	12.5	8.7	11.3	22.3	0.191	0.0	0.0	1.6	6.7	0.102
Bloodletting	0.0	13.0	8.1	15.5	0.302	0.0	4.3	0.0	3.6	**0.050**
Herbal medicine/product	37.5	26.1	43.5	44.0	0.357	12.5	13.0	29.0	20.2	0.304
Massage	62.5	43.5	64.5	64.2	0.295	25.0	21.7	29.0	32.6	0.771
Nutritional supplements	37.5	52.2	61.3	63.2	0.279	25.0	39.1	32.3	37.3	0.510
Prayers/spirituality	87.5	87.0	83.9	81.3	0.829	62.5	78.3	71.0	68.4	0.520

*P* < 0.05 considered to be significant.

**Table 11:** Association between having relatives in the health sector and awareness and use of CAM modalities.

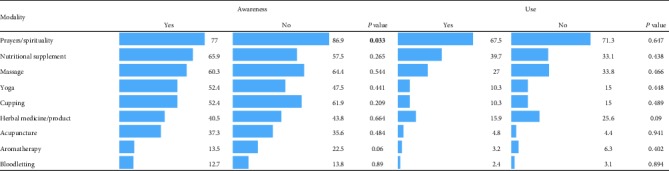

**Table 12:** Association between income and awareness and use of CAM modalities.

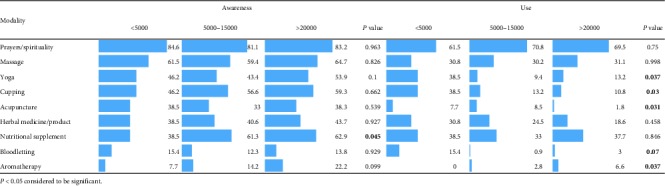

## Data Availability

Data can be obtained upon request from the corresponding author at khanasl@ksauhs.edu.sa.

## Abbreviations

CAM: Complementary and alternative medicine
CAMS: College of Applied Medical Sciences
COM: College of Medicine
CON: College of Nursing
COSHP: College of Science and Health Professions
IRB: Institutional Review Board
KAIMRC: King Abdullah Medical Research Center
KSAU-HS: King Saud bin Abdulaziz University for Health Sciences
MNG-HA: Ministry of National Guard-Health Affairs.

## References

[1] WHO (2002). *WHO Traditional Medicine Strategy 2002–2005*.

[2] Hans M., Martin F. (1954). Study of holarrhena congolensis stapf. from the Gimbi region of the Belgian Congo. *Journal de pharmacie de Belgique*.

[3] National Institutes of Health (2015). *Complementary, Alternative, or Integrative Health: What’s in a Name?*.

[4] Gilani A. H., Rahman A. U. (2005). Trends in ethnopharmocology. *Journal of Ethnopharmacology*.

[5] Tovey P. A., Broom A. F., Chatwin J., Ahmad S., Hafeez M. (2005). Use of traditional, complementary and allopathic medicines in Pakistan by cancer patients. *Rural Remote Health*.

[6] Hollenberg D., Zakus D., Cook T., Xu X. W. (2008). Re-positioning the role of traditional, complementary and alternative medicine as essential health knowledge in global health: do they still have a role to play?. *World Health Population*.

[7] Haq I. (1983). *Medicinal Plants: Report of Committee on Economic And Therapeutic Importance of Medicinal Plants Initiated by the Ministry of Health, Government of Pakistan*.

[8] Hussain S., Dil A. S., Hannan A., Gilani A. H., Bodeker G., Ong C. K., Grundy C. (2005). Islamic republic of Pakistan. *WHO Global Atlas of Traditional, Complementary and Alternative Medicine*.

[9] World Health Organization (2001). *Legal Status of Traditional Medicine and Complementary*.

[10] Al-Faris E. A., Al-Rowais N., Mohamed A. G. (2008). Prevalence and pattern of alternative medicine use: the results of a household survey. *Annals of Saudi Medicine*.

[11] Al-Faris E. A. (2000). The pattern of alternative medicine use among patients attending health centres in a military community in Riyadh. *Journal of Family & Community Medicine*.

[12] AlBedah A. M., Khalil M. K., Elolemy A. T. (2013). The use of and out-of-pocket spending on complementary and alternative medicine in Qassim province, Saudi Arabia. *Annals of Saudi Medicine*.

[13] Mahady G. B. (2001). Global harmonization of herbal health claims. *The Journal of Nutrition*.

[14] Bashir S., Memon R., Gilani A. H. (2011). Antispasmodic and antidiarrheal activities of valeriana hardwickii wall. Rhizome are putatively mediated through calcium channel blockade. *Evidence-Based Complementary and Alternative Medicine*.

[15] Gilani A., Molla N., Rahman A. U., Shah B. H. Phytotherapy—the role of natural products in modern medicine. *Journal of Pharmaceutical and Biomedical Analysis*.

[16] Radi R., Isleem U., Al Omari L., Alimoglu O., Ankarali H., Taha H. (2018). Attitudes and barriers towards using complementary and alternative medicine among university students in Jordan. *Complementary Therapies in Medicine*.

[17] Faqueti A., Tesser C. D. (2018). Use of complementary and alternative medicine in primary healthcare in florianopolis, santa catarina, Brazil: user perception. *Ciência & Saúde Coletiva*.

[18] Bozza C., Gerratana L., Basile D. (2018). Use and perception of complementary and alternative medicine among cancer patients: the CAMEO-PRO study: complementary and alternative medicine in oncology. *Journal of Cancer Research and Clinical Oncology*.

[19] Tsang V. H. M., Lo P. H. W., Lam F. T. (2017). Perception and use of complementary and alternative medicine for low back pain. *Journal of Orthopaedic Surgery*.

[20] Kretchy I. A., Okere H. A., Osafo J., Afrane B., Sarkodie J., Debrah P. (2016). Perceptions of traditional, complementary and alternative medicine among conventional healthcare practitioners in Accra, Ghana: implications for integrative healthcare. *Journal of Integrative Medicine*.

[21] Ghiasuddin A., Wong J., Siu A. M. (2016). Complementary and alternative medicine practices, traditional healing practices, and cultural competency in pediatric oncology in Hawai’i. *Journal of Integrative Medicine*.

[22] Brambila-Tapia A. J., Rios-Gonzalez B. E., Lopez-Barragan L., Saldana-Cruz A. M., Rodriguez-Vazquez K. (2016). Attitudes, knowledge, use, and recommendation of complementary and alternative medicine by health professionals in western Mexico. *Explore*.

[23] Roy V., Gupta M., Ghosh R. K. (2015). Perception, attitude and usage of complementary and alternative medicine among doctors and patients in a tertiary care hospital in India. *Indian Journal of Pharmacology*.

[24] James P. B., Bah A. J. (2014). Awareness, use, attitude and perceived need for complementary and alternative medicine (CAM) education among undergraduate pharmacy students in Sierra Leone: a descriptive cross-sectional survey. *BMC Complementary and Alternative Medicine*.

[25] Asadi-Pooya A. A., Emami M. (2014). Perception and use of complementary and alternative medicine among children and adults with epilepsy: the importance of the decision makers. *Acta Medica Iranica*.

[26] Hussain S., Malik F., Hameed A. (2012). Pakistani pharmacy students’ perception about complementary and alternative medicine. *American Journal of Pharmaceutical Education*.

[27] Hasan S. S., Yong C. S., Babar M. G. (2011). Understanding, perceptions and self-use of complementary and alternative medicine (CAM) among Malaysian pharmacy students. *BMC Complementary and Alternative Medicine*.

[28] Rhode J. M., Patel D. A., Sen A., Schimp V. L., Johnston C. M., Liu J. R. (2008). Perception and use of complementary and alternative medicine among gynecologic oncology care providers. *International Journal of Gynecology & Obstetrics*.

[29] Cochran W. G. (1977). *Sampling Techniques*.

[30] Ashraf M., Saeed H., Saleem Z. (2019). A cross-sectional assessment of knowledge, attitudes and self-perceived effectiveness of complementary and alternative medicine among pharmacy and non-pharmacy university students. *BMC Complementary and Alternative Medicine*.

[31] Gelaw B. K., Tegegne G. T., Bizuye Y. A., Gelaw Y. K. (2014). Assessment of knowledge and attitude of wollo university pharmacy students towards complementary and alternative medicine, north east Ethiopia. *Annals of Ayurvedic Medicine*.

[32] Awad A., Al-Ajmi S., Waheedi M. (2012). Knowledge, perceptions and attitudes toward complementary and alternative therapies among Kuwaiti medical and pharmacy students. *Medical Principles and Practice*.

[33] Ameade E. P. K., Amalba A., Helegbe G. K., Mohammed B. S. (2016). Medical students’ knowledge and attitude towards complementary and alternative medicine–a survey in Ghana. *Journal of Traditional and Complementary Medicine*.

[34] Pokladnikova J., Lie D. (2008). Comparison of attitudes, beliefs, and resource-seeking behavior for CAM among first-and third-year Czech pharmacy students. *American Journal of Pharmaceutical Education*.

[35] Alrowais N. A., Alyousefi N. A. (2017). The prevalence extent of complementary and alternative medicine (CAM) use among Saudis. *Saudi Pharmaceutical Journal*.

[36] Aboushanab T., AlSanad S. (2018). A brief illustration of the official national standards for the safe use of cupping therapy (Hijama) in Saudi Arabia. *Journal of Integrative Medicine*.

[37] Qureshi N. A., Ali G. I., Abushanab T. S. (2017). History of cupping (Hijama): a narrative review of literature. *Journal of Integrative Medicine*.

[38] Doyle C. B., Khan A., Burton N. W. (2019). Recreational physical activity context and type preferences among male and female Emirati university students. *International Health*.

[39] Lee G., Charn T., Chew Z., Ng T. (2004). Complementary and alternative medicine use in patients with chronic diseases in primary care is associated with perceived quality of care and cultural beliefs. *Family Practice*.

[40] Majeed K., Mahmud H., Khawaja H. R., Mansoor S., Masood S., Khimani F. (2007). Complementary and alternative medicine: perceptions of medical students from Pakistan. *Medical Education Online*.

[41] Al Mansour M. A., Al-Bedah A. M., AlRukban M. O. (2015). Medical students’ knowledge, attitude, and practice of complementary and alternative medicine: a pre-and post-exposure survey in Majmaah University, Saudi Arabia. *Advances in Medical Education and Practice*.

[42] Wong L. Y., Toh M. P. H. M., Kong K. H. (2010). Barriers to patient referral for complementary and alternative medicines and its implications on interventions. *Complementary Therapies in Medicine*.

[43] Zimmerman C., Kandiah J. (2012). A pilot study to assess students’ perceptions, familiarity, and knowledge in the use of complementary and alternative herbal supplements in health promotion. *Alternative Therapies in Health and Medicine*.

[44] Jamshed S. Q., Khan M. U., Ahmad A., Elkalmi R. M. (2016). Knowledge, perceptions, and attitudes toward complementary and alternative medicines among pharmacy students of a Malaysian Public University. *Journal of Pharmacy & Bioallied Sciences*.

[45] Kanadiya M. K., Klein G., Shubrook J. H. (2012). Use of and attitudes toward complementary and alternative medicine among osteopathic medical students. *The Journal of the American Osteopathic Association*.

[46] Schnabel K., Binting S., Witt C. M., Teut M. (2014). Use of complementary and alternative medicine by older adults—a cross-sectional survey. *BMC Geriatrics*.

[47] Samara A. M., Barabra E. R., Quzaih H. N., Zyoud S. H. (2019). Use and acceptance of complementary and alternative medicine among medical students: a cross sectional study from Palestine. *BMC Complementary and Alternative Medicine*.

